# Patient Attitudes towards Physician Nonverbal Behaviors during Consultancy: Result from a Developing Country

**DOI:** 10.1155/2014/473654

**Published:** 2014-02-04

**Authors:** Fahad Hanif Khan, Raheela Hanif, Rumina Tabassum, Waris Qidwai, Kashmira Nanji

**Affiliations:** ^1^Medicine, Civil Hospital, B-103, Block 14, Gulistan-e-Johar, P.O. Box 75290, Karachi 74200, Pakistan; ^2^Internal Medicine, Aga Khan University Hospital, Karachi 74800, Pakistan; ^3^Obstetrics and Gynaecology, Dow International Medical College, Karachi 74200, Pakistan; ^4^Family Medicine Department, Aga Khan University Hospital, Karachi 74800, Pakistan

## Abstract

*Background*. Nonverbal behaviors have a significant impact on patients during consultations. This study was undertaken to find out the attitudes and preferences of the patients regarding nonverbal communication during consultations with physicians, in a tertiary care hospital. *Methods*. A questionnaire based cross-sectional study was carried out at the Aga Khan University Hospital, Karachi, Pakistan, during the months of January to March 2012. All patients (>18 years of age) coming for consultancy in the family medicine clinics were approached; out of 133, 120 agreed to participate. The subjects were asked questions regarding physician's comforting touch and eye contact and their responses were noted. The data were analyzed using SPSS and chi-square test was used to identify corelations. *Results*. Overall, 120 patients were enrolled. About 58.3% were men and 41.7% were women with a mean age of 34.9 ± 10.9 years. 95.8% were Muslims and 57.6% had more than 12 years of education. Among females 74% wanted supportive touch from doctors, used to comfort the patient (45%) or to show respect (27.5%) or as healing (30%). 86.1% of the respondents believe that establishing eye contact with the patient shows that the doctor is attentive towards his/her patient. The eye contact should be brief but regular (54.1%) and prolonged staring (36.7%) makes them uncomfortable. *Conclusion*. Nonverbal communication helps to strengthen the doctor-patient relation as patients do appreciate positive touch and eye contact from their physicians.

## 1. Introduction

Establishing good communication, either verbal or nonverbal, with patient is an essential and important component to develop a good doctor-patient relation. Numerous studies have explored the mechanisms and importance of nonverbal communications during medical interviews [1]. Face-to-face interaction (including facial expressions and eye contact), expressive touch, body language, paralinguistics (vocal communication which is discrete from actual language), interpersonal proximity, physical appearance, and eloquent gestures all make verbal conversation more expressive and meaningful [2]. Evidence shows that physician's nonverbal behavior leads to higher patient satisfaction, but this is affected by a number of factors, including gender of the doctor as well as of the patient [3, 4]. A study, from Switzerland, showed both male and female doctors should display different set of nonverbal behavior to maximize patients satisfaction [5].

Nonverbal communication has been shown to be important in dealing with pediatric age groups [6] and with those recovering from disabilities [7–9]. Eye contact and physical touch are commonly used as effective tools in nonverbal communication [1, 10]. Touch can be perceived as comforting and healing [11]. General practitioners may feel reluctant to use touch other than procedural touch, because of the fear of misinterpretation of such behavior however, many patients believe that, particularly in distressing situations, expressive touch is acceptable [12]. A study conducted in Canada on female patients concluded that, as compared to males, females were more tolerant of comforting touch [10]. Eye contact is another important nonverbal behavior and is especially essential for building good rapport with elderly individuals [13]. It is mostly taken as a sign of respect, care, and attention from a doctor [1]. However, if eye contact is coupled with attentive listening it inclines the interaction towards more patient-centered communication [14]. Nowadays, the use of computers and especially the electronic health records (HER), during medical interviews, is a big obstacle in using eye contact as an effective way to communicate [15].

We therefore directed our study to explore the expectations and perceptions of patients, in a developing Asian country, regarding touch and eye contact by physicians during consultancy, as most of studies on nonverbal communication were conducted in West and we consider that responses in our part of the world would differ.

## 2. Methods

This cross-sectional study was conducted at the Community Health Centre (Family Practice Clinic), at the Aga Khan University Hospital, Karachi, Pakistan (AKUH), during the months of January to March 2012. AKUH is one of the major, tertiary care teaching hospitals in Karachi, with state-of-the-art Family Practice Clinics. On an average, 200 family practice patients with mostly primary and secondary care level problems were seen daily by 12–16 family physicians at the clinics. These clinics were chosen to obtain a diverse sample belonging to various socioeconomic strata.

An interview based questionnaire was developed by the researchers through literature search (for validated questions), input from colleagues and from experts in the field of family medicine. The questionnaire was then translated from English version to Urdu language and was back translated in English to check for reliability and any discrepancies found were removed. The questionnaire was pretested on 10 participants (data not included in the results) before finalizing it.

Interviewers (two medical graduates) were trained for data collection and on the use of the questionnaire to ensure uniformity of application. The questions were asked in English and in Urdu depending on the participant's ease with the language. Patients were chosen randomly and interviewed in the waiting area, before their consultancy with the physician, irrespective of their age, gender, educational status, and the reason for their visit. Those who were willing to participate did not receive any monetary compensation and were asked to sign a written consent form and the provision of confidentiality was ensured to them.

The questionnaire was composed of two sections. Section 1 included demographics like age, gender, religion, education, marital status, and occupation. In section 2 the questions were focused on the nonverbal communication modes of touch and eye contact only, exploring the patient's expectations about willingness, comfort, and perceptions regarding the two modalities. Other questions were directed to know which part of the body they would allow the physician to touch and the duration of eye contact. The study was reviewed and approved by the Dow University of Health Sciences, Karachi.

The data was entered on Statistical Package for Social Sciences (SPSS) version 16. Proportions were calculated for all the variables of interest and chi-square test was used to assess relations of gender and educational level with nonverbal communication modalities. A *P*-value of <0.05 was considered statistically significant throughout the study.

## 3. Results

A total of 133 patients were approached, out of which 120 patients agreed to participate and were interviewed. The mean age of the participants was 34.9 ± 10.9 years, out of which 32.5% were between 20–30 years of age and 39.2% were 30–40 years. The male (58.3%) to female (41.7%) ratio was 1.4. Eighty-three respondents were married with 80.1% (96) having grade ten or more education (Table 1).

**Figure 1:**
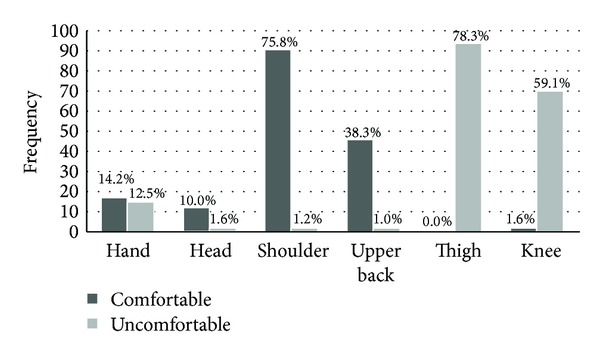
Frequencies and comfort level when touched on different body parts (*n* = 220).

In the study 62.6% (62) males and 37.3% (37) females formed a total of 99 participants who stated that a physician should use touch during their visits (*P*-value: 0.03) (Table 2); however there was no statistical significance in relation to their educational level (*P*-value: 0.28), religious beliefs (*P*-value: 0.63), and marital status (*P*-value: 0.61). About 75.8% of 120 patients said that they can be tapped at their shoulder, 38.3% on their upper back, and 14.2% on their hands; however 59.1% do not want to be touched on either knee (Figure 1). If tapped on their shoulder, 35.8% would take it as a gesture of comfort and 24.1% would take as respect followed by 21.6% as healing and 19.1% as a way to increase mutual understanding (*P*-value: 0.001) (Table 3).

The desire to have eye contact was not statistically significant to gender (*P*-value: 0.08), religion followed, or literacy level. However, 95.8% (115) patients felt comfortable if eye contact was established by the clinician in order to develop the patient-doctor relation (Table 2). Eighty-six percent of the respondents felt that this is a sign that the doctor is paying attention to their complaints. Forty-six percent felt that this was done to gain confidence in communication. According to 54.1%, eye contact should be brief but regular rather than only when patient is talking about symptoms (1.7%). Long stares (36.7%) and objects in the surrounding environment, like computers (19.1%) make them uncomfortable during eye contact (Table 4).

## 4. Discussion

This is a pioneer study from the South Asian region to assess patient's attitudes towards nonverbal communication through touch and eyecontact during consultations. The outcomes from this study should reassure many medical practitioners regarding the use of comforting touch to patients [12], as 82.5% of respondents wanted sympathetic touch, especially in distressing situations.

Touch influences right from a doctor's greetings and simple hand shake [1]. However, as opposed to more distal touch [11], this study reveals that touch on the shoulder (75.8%) or upper back (38.3%) is more acceptable, when compared to touching on the hands (14.2%). This perhaps reflects the beliefs and religious obligations in a Muslim society. Yet there exists some gender bias, as patients (of both sexes) are more comfortable to touch from a female doctor as compared to that from a male [11]. Our results are consistent with study by Street and Buller [16], who studied the nonverbal communication in patient-doctor interactions and they also found no statistically significant correlation between the level of education of patients and their perceptions regarding physician's touch, whereas a study from John Hopkins has shown contrasting results that physician's touch can be “dominating or controlling” to people [17], but as our results highlight it is also taken as a gesture of comfort or respect and as healing, which is in agreement with Osmun et al. [11].

A good physician begins to care for the patient as soon as he/she looks at him. In this present study, 86.1% of the patients eagerly wanted the doctor's attention through his/her eye contact as pointed out by Marcinowicz et al. [1]. Even a simple gesture of frowning can have a positive impact on the patient's satisfaction [18]. We also found that in the opinion of 54.1% participants, a regular but brief visual interaction is more effective, rather than long durations of eye contact [14].

The use of the computer for keeping electronic records has been a hindrance in developing a good patient-doctor relation, as in this study 19.1% people are distracted from objects in the clinical environment and feel that less attention is being directed to them. This has been acknowledged by many general practitioners (GPs) as there was a drastic decrease (from 2001 to 2008) in the use of computer by GPs during consultancy [19]. Observing the patient together with listening and informative responses makes a medical interview more patient centered and thus results in better therapeutic outcome [20].

This research is a first step in exploring the importance of nonverbal communications among physicians in the South Asian region, but it does have many limitations. First, the study was conducted at a single, privately governed hospital and needs a larger sample size to generalize the results to all private or to public sector hospitals. Secondly, only two nonverbal modalities have been evaluated in this study, rather than exploring all nonverbal means of communication. Third, the attitudes and perceptions of physicians regarding nonverbal modalities were not assessed. Fourth, online literature search revealed a study by Stepanikova et al. [4] who concluded that racial backgrounds do play a role in influencing nonverbal communication and this aspect is not covered in the present study.

## 5. Conclusion

Positive, effective, and sensitive nonverbal behavior helps to strengthen the doctor-patient bond. This study does require further clarification and elaborations, but the results do demonstrate the importance of touch and eye contact during the physician's consultancy. Patients do require, from their doctors, a comforting touch on shoulder and regular but brief eye contacts to demonstrate his/her attention towards the patients.

We believe further research on this important subject, which could be multicentric, should be further explored, with larger sample populations and covering all aspects of nonverbal communication. Moreover, researches investigating physician's perspective regarding nonverbal means of communication during consultancy are warranted to enhance the importance of this ignored yet eminent topic.

*Annex: Questionnaire*


Name (optional):Age:Gender: Male FemaleReligion:Education: Illiterate Can read and write Primary Secondary (grade VIII) Grade 10 Higher secondary Graduate PostgraduateMarital Status: Married UnmarriedOccupation: Private Job Government job Self-employed Retired


*Consent*

The following questions are designed to assess the preferences for touch and eye contact during consultations.

Participation is completely voluntary. You may decide not to participate or if you decide to participate, you are free to withdraw from the study at any time.

Your confidentiality is assured. Results of the survey will only be reported in the aggregates; individuals will not be identified in any way in the reports. Your data will be coded under a random identifier that cannot be linked to you.

Thanking you for your co-operation.

“I agree to voluntary participate in this study”
Signature: ———    Date: ———(1) Do you want physical touching of your body by doctor during consultation? (a) Yes (b) No (c) Don't know(2) Do you approve physical touching of your body by doctor during consultation? (a) Yes (b) No (c) Don't know(3) Do you feel comfortable with physical touching of your body by doctor during consultation? (a) Yes (b) No (c) Don't know(4) You would take a touch from a doctor during consultation as a gesture of? (a) Empathy (b) Cure (c) Respect (d) Comfort (e) Communication with doctor (f) Other ———(5) Which part of your body you would comfortable with? (a) Hand (b) Head (c) Shoulder (d) Knee (e) Upper back (f) Other ———(6) Which part of your body you will not be comfortable with? (a) Abdomen (b) Thigh (c) Others ———(7) Do you feel comfortable with his/her eye contact? (a) Yes (b) No (c) Don't know(8) For how long should an eye contact be? (a) Should be throughout consultation (b) Should be brief but regular (c) Should be more at beginning less in the end (d) Should be more at endless in the beginning (e) Others ———(9) What do you feel when a doctor makes an eye contact? (a) Confidence in yourself (b) Confidence in communication (c) Secure (d) Attended (e) Much more important (f) Other ———(10) What are the things which would make you uncomfortable during eye contact? (a) Long stare (b) Smiling eyes (c) Frequent blinking (d) Others———(11) What do you feel if a doctor does not make an eye contact? (a) Religious (b) Less confident (c) Short attention span (d) Liar (e) Lack of interest


## Figures and Tables

**Table 1:** Demographic profile of the participants (*n* = 120).

Age (mean ± SD)	34.9 ± 10.9	
	*n*	%

Gender		
Male	70	58.3
Female	50	41.7
Education		
Illiterate	8	6.6
Below grade 10	16	13.3
Grade 10 and above	96	80.1
Marital Status		
Married	83	69.1
Unmarried	37	30.8
Occupation		
Employed	83	69.1
Students/retired	14	11.6
Housewife	23	19.2

**Table 2:** Gender specific need of expressive touch and eye contact from the physician during consultancy.

	Gender			*P* value*
	Male *n* (%)	Female *n* (%)	Total *n* (%)	
Do you want physical touching by the doctor?				
Yes *n* (%)	62 (88.5)	37 (74)	99 (82.5)	0.03
No *n* (%)	8 (11.5)	13 (26)	21 (17.5)	
Total *n* (%)	**70 (100)**	**50 (100)**	**120 (100)**	
Do you feel comfortable with his/her eye contact?				
Yes *n* (%)	87 (95.6)	28 (96.5)	115 (95.8)	0.08
No *n* (%)	4 (4.3)	1 (3.4)	5 (4.1)	
Total *n* (%)	**91 (100)**	**29 (100)**	**120 (100)**	

*Chi-square test.

**Table 3:** Perception of patients regarding touch body parts (*n* = 120).

	You would take a touch from a doctor during consultation as a gesture of?				
	Empathy *n* (%)	Healing *n* (%)	Respect *n* (%)	Comfort *n* (%)	Mutual understanding *n* (%)
Which part of the body you would be comfortable with?					
Hand	3 (2.5)	8 (6.6)	3 (2.5)	8 (6.6)	4 (3.3)
Head	2 (1.6)	4 (3.3)	4 (3.3)	5 (4.1)	2 (1.6)
Shoulder	17 (14.1)	26 (21.6)	29 (24.1)	43 (35.8)	23 (19.1)
Upper back	10 (8.3)	10 (8.3)	14 (11.6)	18 (15)	15 (12.5)

*P*-value: 0.001 (chi-square test).

**Table 4:** Expectations and perceptions of patients regarding eye contact (*n* = 120).

S. no.	Statements	*N*	%
1	Duration of eye contact		
	Should be throughout consultation	24	20
	Should be brief but regular	65	54.1
	Should be more at beginning less in the end	26	21.7
	Should be more at the end less in the beginning	3	2.5
	When talking about symptoms	2	1.7

2	Feeling when a doctor makes an eye contact		
	Confidence in myself	11	9.2
	Confidence in communication	55	45.8
	Secure	16	13.3
	Attended	107	86.1
	It is a bad behavior by a doctor	1	0.8

3	Acts/objects which make uncomfortable during eye contact		
	Long stare	44	36.7
	Smiling eyes	8	6.7
	Frequent blinking	13	10.8
	Roaming eyes	2	1.7
	Items in the surrounding	23	19.1
	None	20	16.6

Redundant categories have been removed.
